# Electrotactile Communication via Matrix Electrode Placed on the Torso Using Fast Calibration, and Static vs. Dynamic Encoding

**DOI:** 10.3390/s22197658

**Published:** 2022-10-09

**Authors:** Jovana Malešević, Miloš Kostić, Fabricio A. Jure, Erika G. Spaich, Strahinja Došen, Vojin Ilić, Goran Bijelić, Matija Štrbac

**Affiliations:** 1 Tecnalia Serbia, Ltd., 11000 Belgrade, Serbia; 2 Neurorehabilitation Systems, Department of Health Science and Technology, Faculty of Medicine, Aalborg University, 9220 Aalborg, Denmark; 3 Department of Computing and Control Engineering, Faculty of Technical Sciences, University of Novi Sad, 21102 Novi Sad, Serbia; 4 Tecnalia, Basque Research and Technology Alliance (BRTA), 20009 Donostia-San Sebastian, Spain

**Keywords:** haptics, tactile communication, feedback coding, multi-pad electrode

## Abstract

Electrotactile stimulation is a technology that reproducibly elicits tactile sensations and can be used as an alternative channel to communicate information to the user. The presented work is a part of an effort to develop this technology into an unobtrusive communication tool for first responders. In this study, the aim was to compare the success rate (SR) between discriminating stimulation at six spatial locations (static encoding) and recognizing six spatio-temporal patterns where pads are activated sequentially in a predetermined order (dynamic encoding). Additionally, a procedure for a fast amplitude calibration, that includes a semi-automated initialization and an optional manual adjustment, was employed and evaluated. Twenty subjects, including twelve first responders, participated in the study. The electrode comprising the 3 × 2 matrix of pads was placed on the lateral torso. The results showed that high SRs could be achieved for both types of message encoding after a short learning phase; however, the dynamic approach led to a statistically significant improvement in messages recognition (SR of 93.3%), compared to static stimulation (SR of 83.3%). The proposed calibration procedure was also effective since in 83.8% of the cases the subjects did not need to adjust the stimulation amplitude manually.

## 1. Introduction

Electrotactile stimulation is a well-known and widely used method of tactile communication that has advantages over other means of haptic feedback due to its affordable price, compact size, low weight, fast response and efficient power consumption [1]. In this approach, low-intensity electrical pulses are injected into the skin to activate cutaneous afferent fibers [2] and elicit sensations such as pressure, vibration, tingling or tickling. The sensation quality and quantity can be modulated by changing the stimulation parameters (intensity and/or frequency), which can be used to communicate temporal and spatial information to the user, e.g., by associating sensation patterns to the specific messages [3,4]. This approach may be most advantageous in applications where other sensory channels such as auditory [5,6] or visual [7,8] are restricted or are already overwhelmed. It was demonstrated to be an intuitive and simple method to convey tactile and/or proprioceptive information on the status of the impaired limbs [9,10,11] or upper [12] and lower [13] limb myoelectric prostheses. More recently, it has also been recognized as a promising approach for providing tactile information in teleoperation scenarios [14] and as an additional feedback channel in virtual environments [15]. Similarly, electrotactile stimulation is of particular interest within the SIXTHSENSE project [16], where the goal is to develop the next generation of wearable health monitoring systems aiming to enhance the situational awareness of first responders, while ensuring their undivided auditory and visual attention.

A major drawback and a current barrier to a wider practical use of electrotactile stimulation systems is the need for system calibration before every use, which could be tedious and time consuming, especially when using multi-channel interfaces. Here, stimulation parameters need to be adjusted individually for each channel to avoid imperceivable, uncomfortable or even painful sensations [17,18,19]. A simple approach to bypass these shortcomings in certain applications is to apply mechanical stimulation by directly activating mechanoreceptors through piezoelectric, pneumatic, hydraulic, electromagnetic or vibration motors [20,21]. However, the mechanical actuators rely on the physical movement of mechanical components that are often rigid and hefty, which limits their integration, and these movements can be hard to perceive during intense physical activity [22,23,24]. In many scenarios, this is not favorable. For instance, when the tactile information is transmitted to the first responders during diverse rescue missions or firefighting, it is of paramount importance that the communication is clearly perceivable during intense physical activity while not obstructing their body movements or ability to carry victims or equipment [25]. Furthermore, due to the large morphological differences across subjects, forming and fitting a garment embedded haptic display to the user’s body to achieve both comfort and functionality is a significant design challenge [26]. Therefore, a thin, lightweight, soft and skin-adhering device that can be produced using electrotactile technology represent a promising direction to implement a feedback channel for first responders. Recent technological advances such as flexible memory devices [27], Bipolar Junction Transistor-based biosensors [28] and graphene-based humidity sensors [29] are promising in terms of further device miniaturization and garment integration, as well as the potential design of integrated closed loop systems. However, as explained before, the calibration is a drawback of this technology, and a significant effort was dedicated to simplifying the electrotactile calibration procedures [30,31].

In addition to positive physical characteristics, another benefit of electrotactile stimulation is a high bandwidth of information transmission. The stimulation parameters including the pulse amplitude, pulse width, frequency and, in case of multi-point systems, stimulation location can be dynamically and independently modulated [4,9,32,33]. The pulse width and amplitude affect the intensity of the elicited sensation. To obtain a perceivable but not uncomfortable sensation, these parameters need to be in the range between the sensation (ST) and the discomfort threshold, which are subject-specific and time-variable [2,34], but can be determined through calibration [28]. The effective bandwidth of electrotactile communication channel will depend on the system specification (e.g., parameter resolution and number of stimulation points) as well as the perceptual capacities of the subject that can be assessed by psychometric measurements (i.e., just-noticeable differences, two-point discrimination threshold, etc.) [35].

Multi-point tactile stimulation systems can increase communication bandwidth by transmitting information through the location of the stimuli. The selection of the number, sizes, shapes, and arrangement of the stimulation pads depends on the size of the targeted area of the body and underlying fat layer [36,37]. The fingertips have the highest tactile acuity within the human body [38,39,40], but the fingertips are not the optimal location for applying tactile stimulation in every scenario, especially in those that include the extensive use of the hands. Faced with similar challenges, a number of different wearable tactile displays was designed for application on the torso [41,42,43,44,45,46]. Although the torso has one of the lowest spatial resolution for two-points discrimination [36], this is compensated by a large area that can be exploited for stimulation. In addition, to ensure the hands-free information transfer [47,48], it has been shown that the tactile display placed on the torso can allow high-performance communication when electrode pad size, arrangement and patterns are selected properly [49].

The stimulation points could be leveraged for information transfer both statically and dynamically. In static modes, a message can be encoded by the spatial activation of single pad or by the simultaneous activation/ deactivation of a group of pads. In dynamic modes, the information can be transmitted through sequential activation of singe pads or pad subsets. This approach allows “drawing” spatio-temporal patterns onto the user’s body [50]. With a matrix arrangement of the pads, the special signs—“letters”—directions or motion illusions are simple to implement. By using only a few pads, a large number of such spatial patterns can be presented enabling the encoding and transmission of a large number of tactile messages. These techniques have been used to increase the recognizability of the information presented in the form of Chinese and English characters [51,52], shapes [53], and military hand signs [49]. In our recent publication several methods for the dynamic presentation of the spatial patterns have been tested with promising results [54]. However, such dynamic encoding has not been directly compared to a static single pad encoding, which is the simplest approach to convey information.

In the present study, we therefore compared the two methods of tactile communication, namely, static and dynamic approach, using a 3 × 2 multi-pad electrode positioned on the lateral side of the torso. To this aim, the success rate (SR) of discriminating between six individually activated pads (static method) was contrasted with the discrimination of six predefined spatial movement patterns (dynamic method). Additionally, a fast approach for the calibration of stimulation intensity was investigated by assessing the relation between the calibrated pulse amplitude and the sensation threshold. Importantly, the recruited subjects included a group of first responders, who are the target users of the electrotactile interface, and hence, the present study is also the first assessment of the proposed approach (electrode design and encoding) in the target population.

## 2. Materials and Methods

### 2.1. Hardware Ccmponents

**Figure 1:**
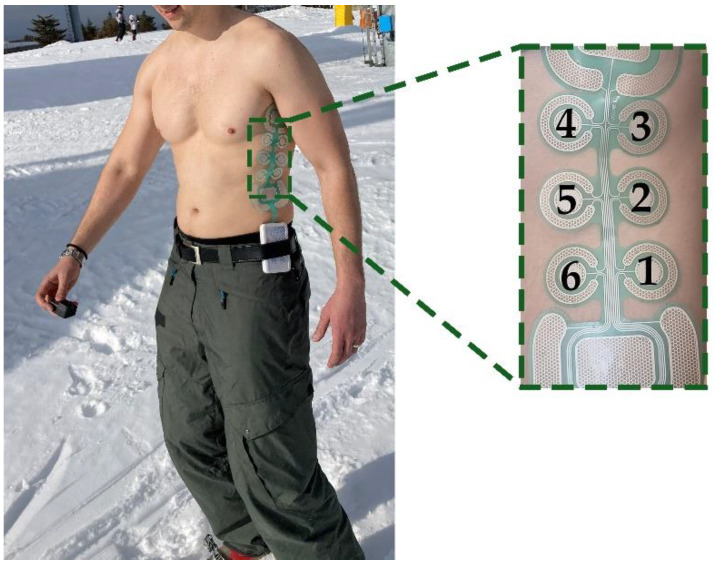
The electrode with pad enumeration placed on the left lateral side of the torso of a first responder (mountain rescuer).

Experimental setup comprised a custom multi-pad electrode and a multi-channel stimulation module (Alpha Mobile Device, Global Electronic Solutions, Novi Sad, RS), both developed within the SIXTHSENSE project [16]. The electrode was designed based on the project requirements. The preliminary psychometric evaluation of this system confirmed that this design choice was suitable for the application of interest [54,55,56]. The electrode design considered six smaller circle-shaped pads and two larger pads with rounded rectangle shape (Figure 1). Based on previous pilot studies [55], we decided that type of feedback and type of coding should be separated between these two pad types, i.e., 2 large pads will be used to transmit increase/decrease in the certain variable via frequency modulation, while 6 smaller pads will be used to transmit some specific command via spatial modulation. Hence, in this study only 6 central pads were used.

The circular pads in 3 × 2 configurations had an 8.5 mm radius with a 45 mm center-to-center distance between the pads. A distributed reference pad with a total surface of 445 mm^2^, surrounded each pad of the electrode in a quasi-concentric configuration with a small opening gap to route the leads of the inner pad. 

The current-controlled multi-channel pulse generator within the Alpha Mobile Device was used to produce symmetric biphasic rectangular stimulation pulses. The pulse amplitude could be adjusted between 0 mA and 9 mA in steps of 0.1 mA for each channel individually. The stimulation frequency and pulse width were set to 50 Hz and 300 µs, respectively.

### 2.2. Protocol

#### 2.2.1. Subjects

Twenty healthy subjects participated in the study, 13 males and 7 females, age range between 23 and 38 years. Twelve subjects (nine males and three females) were active first responders from the Mountain Rescue Service of Serbia. Each subject signed an informed consent form. The study was conducted following the Declaration of Helsinki and the experimental protocol was approved by the local ethics committee (number 1322/III-19, date 17 March 2021).

#### 2.2.2. Setup

The electrode was positioned on the left side of the torso and secured with a stretchable girdle while the subject was standing. It was aligned with the midaxillary line, to have three pads on the ventral and three on the dorsal side of the torso, following the protocols established in our previous research [55]. The subjects’ responses were collected using a dedicated custom-made LabVIEW application (National Instruments, Austin, TX, USA) installed on a touch-screen tablet PC that was positioned in front of the subject on an elevated table to enable the interaction with minimal movements of the torso. The test was conducted indoors.

The protocol included two tests in which the task was to identify six tactile messages that were encoded using two different methods. The first test investigated a straightforward encoding approach in which a message was associated with a pad of the electrode, which is a common method described in the literature [57,58,59]. This test assessed the subjects’ ability to discriminate a single active pad within the multi-pad electrode, i.e., the static recognition of stimuli location (spatial discrimination). In the second test, the messages were associated with specific moving sensations. The subjects were asked to identify the pattern’s movement of the active pad, i.e., the recognition of dynamic stimulation patterns (movement discrimination). The two tests were matched in duration and number of messages. Both tests consisted of four phases: amplitude calibration, familiarization, reinforced learning and validation.

In the calibration phase, the pulse amplitude for each of the six pads was individually adjusted following the described procedure. The procedure initiated by highlighting the active pad to be calibrated on the electrode representation on the screen. The stimulation started with the pulse amplitude of 0.6 mA, which was automatically increased by 0.1 mA every 400 ms. The current amplitude was not displayed to the subject. The subjects were instructed to indicate when they first perceived the stimulus, i.e., ST, by pressing the appropriate button on the screen. After reaching the ST, the amplitude was doubled, and this was deemed to represent the localization amplitude (LA), according to our previous work [28]. The LA is the amplitude that elicits a clearly perceivable sensation that is localized to the area below the active pad. In case that the doubled ST amplitude for a specific pad causes uncomfortable sensations, muscle contractions or weak or unclear sensations, the subjects had the option to manually adjust the intensity by pressing the “+” or “−” buttons on the screen. The final chosen amplitude was then adopted as the LA for that pad. This process was performed in 240 individual pad calibrations (20 subjects × 6 pads × 2 tests).

**Figure 2:**
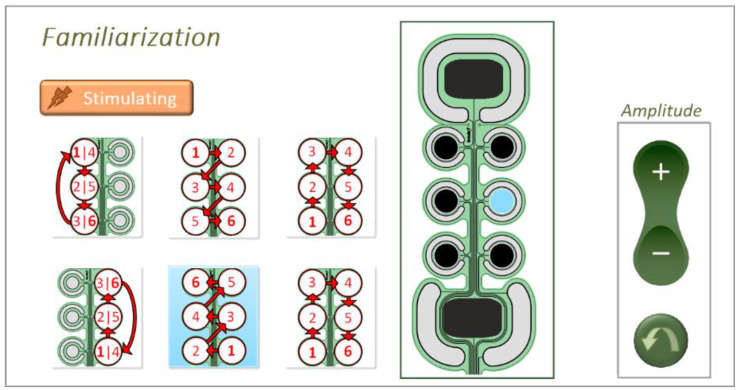
Familiarization phase during the pattern recognition test. Active pattern with activation order (**left**) and currently active pad (**middle**) are highlighted on the screen in blue color. This is the GUI seen by the subjects and the controls in the amplitude box were used to adjust the stimulation intensity, as described in the text.

The experimental procedure in familiarization, reinforced learning and validation phase was the same in both static (pad recognition) and dynamic test (pattern recognition). During the familiarization phase, pads/patterns in the spatial discrimination/ movement recognition tests were activated in a pseudo-random order. Simultaneously, the active pad/movement pattern was presented to the subjects on the electrode sketch on the screen. In the movement recognition test, in addition to the drawing of the pattern (Figure 2, left panel, blue shading), the currently active pad was displayed on the electrode sketch (Figure 2, middle panel, blue pad) to facilitate the mental mapping between the experienced sensation and the position of the active pad. In the static test, a single pad was active for 3 s, while in the dynamic test, each pad was active for 0.5 s, hence, 3 s for the whole 6-pad pattern. The pad/pattern activation was repeated two times, separated by a one-second pause.

In the reinforced learning phase, the visual feedback was removed, the stimulation (pad/pattern) was delivered two times for 3 s and the subjects were asked to identify the presented pad/pattern by indicating it on the screen. If the answer was correct, the pad/pattern was marked with a green frame. Otherwise, the correct pad/pattern was indicated with a red frame. The visual feedback of the correct answer lasted for 3 s. One second after the end of the visual indication, the next pad/pattern was activated, and the process was repeated.

Finally, the subjects performed the validation phase. The validation procedure was the same as the reinforced learning phase with the exception that, in this case, the subjects did not receive visual feedback on the correctness of their answers. In both the reinforced learning and validation phases, each pad/pattern was repeated five times in a pseudo-random order.

Although the amplitude of an individual pad could only be changed in the calibration phase, it should be noted that in the other phases, the subject had an option to increase or decrease stimulation intensity on all pads simultaneously. More specifically, when the subject pressed an up/down button on the GUI, the intensity of all pads was increased/decreased by a fixed factor (i.e., 0.2 mA). This was introduced to mimic the envisioned field trials need of first responders, where the calibration (longer process) would be performed only once after donning the device, while the users could later ramp up or down the overall intensity (fast adjustment). Considering the stationary setup and the short duration of the tests described here, this option was rarely used.

The order of the tests was pseudo-randomized to avoid training effects; hence, half of the subjects started with the pad discrimination, whereas the other half started with the pattern recognition test. There was 10 min pause between the two tests, and during that time, the girdle with the electrode stayed on the subjects. The subjects needed between 8 and 19 min (mean 12.4 min) per test, and the total duration of the session lasted less than 45 min per subject. The pause was introduced to ensure equal initial conditions for both tests, as continuous use of electrostimulation was reported to cause sensory adaptation [60], especially in subjects highly sensitive to electrical stimulation; however, the recovery occurs within minutes [61].

### 2.3. Movement Patterns

**Figure 3:**
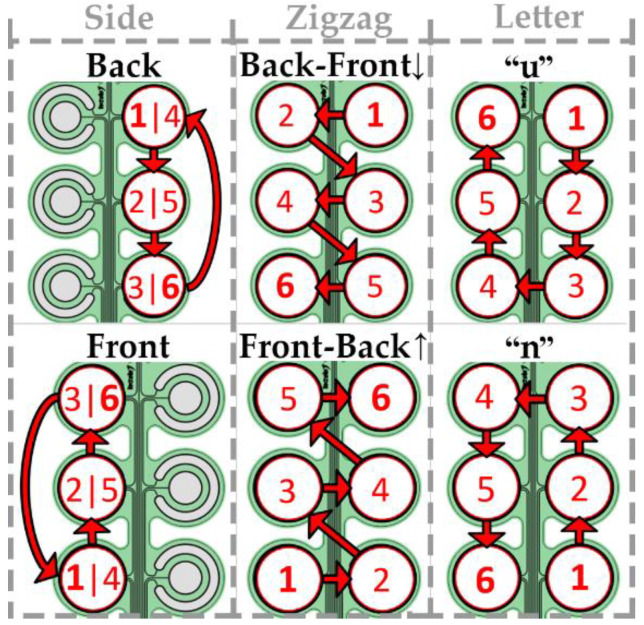
Six movement patterns used in the study. The numbers indicate the order in which the pads were activated. Pads on the right side of the electrode drawing were positioned on the dorsal side of the torso.

To match the number of the electrode pads used in the spatial discrimination test, six movement patterns were defined. A pattern represented the sequential activation of six pads for half a second (i.e., 3 s per pattern). The patterns were divided into three groups, “side”, “zigzag” and “letter”, according to the type of the movement “trajectory”, and each group included two variations of the movement pattern, as illustrated in Figure 3. The patterns were selected to represent movements in which both shape and direction can be used as cues maximizing the discriminability for the envisioned future applications. 

In the “side” group, the pads were activated along the dorsal side of the torso following the downward sequence or along the ventral side of the torso upwards. The sequence was repeated twice without pause (six pad activations in total).

In the “zigzag” group, the sequence was selected to activate alternately two neighboring pads on the opposite side of the torso, while moving downward (staring from pad #3, Figure 1) or upward (starting from pad #6, Figure 1).

The patterns from the “letter” group comprised a sequence of three activations of the pads on the dorsal side followed by three activations on the ventral side of the torso, forming letters “u” (starting from pad #3, Figure 1) and “n” (starting from pad #1, Figure 1).

### 2.4. Data Analysis

The main outcome measure of the study was the SR defined as the percentage of correct identifications of (1) the individual activated pads in the spatial discrimination test and (2) the dynamic patterns in the pattern recognition experiment. A non-parametric Wilcoxon signed-rank test was used to compare the SR between the two assessments (normality assessed with Kolmogorov–Smirnov test). The confusion matrices were used for the visualization of correctly and wrongly observations estimated for pads and movement patterns.

The calibration was performed two times for each electrode pad, once for each test. The outcome measure of the calibration process was the appropriateness of the quick estimation of the LA obtained by doubling the ST. This was measured by counting the number of manual LA adjustments. If the LA was not adjusted by the subject, the automatically estimated LA amplitude was considered satisfactory, which is the desired outcome and confirms the effectiveness of the proposed method.

## 3. Results

### 3.1. Spatial versus Movement Discrimination

**Figure 4:**
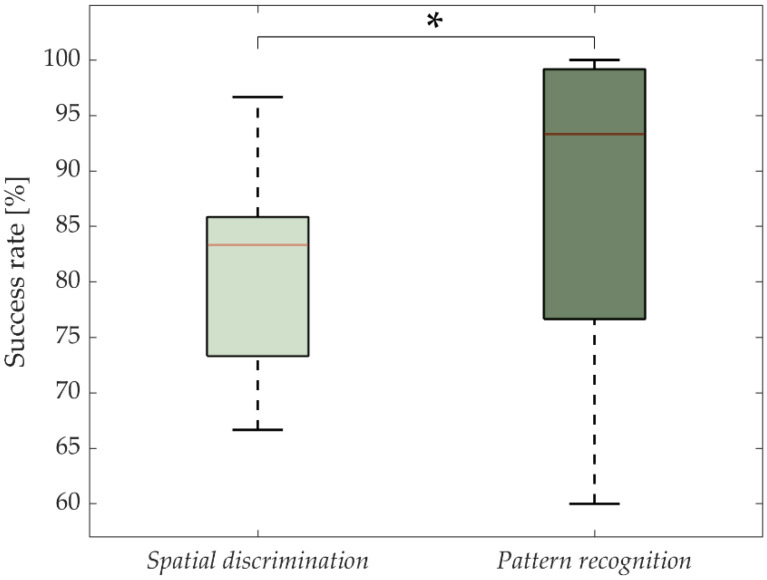
Success rate in spatial discrimination of a single pad within the six-pad electrode (static encoding) versus movement pattern recognition (dynamic encoding). The red lines and boxes indicate the median and interquartile range, respectively. The asterisk indicates significant differences (*: *p* < 0.05).

The overall results of the spatial discrimination of six pads within the multi-pad electrode versus movement pattern recognition are presented in Figure 4.

The statistical analysis showed a significant effect between the two identification methods (*p* = 0.03), the movement pattern SR being significantly higher than the single-pad spatial discrimination. The SR (medians/IQRs) were 83.3/12.5% and 93.3/22.5% for a single-pad spatial discrimination and pattern movement recognition, respectively. Six subjects achieved a maximal SR during pattern recognition, whereas none of the subjects was able to achieve 100% during the recognition of the static activations of the single pads.

**Figure 5:**
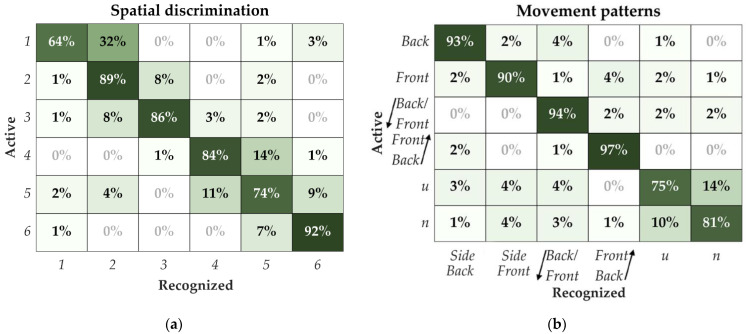
Confusion matrices for (**a**) spatial discrimination of the pads and (**b**) movement pattern recognition. The rows indicate the pad/pattern which was delivered, and the columns report pad/pattern that the subject selected (recognized).

The confusion matrix for pad discrimination is shown in Figure 5a. The highest recognition rate (92%) was achieved for pad #6 (Figure 1) positioned on the bottom row of the dorsal side of the torso. On the other hand, pad #1, which was also in the bottom row but on the ventral portion of the torso, was the most difficult to identify correctly (64%). The most common error in the recognition was confusing the active pad for its direct neighbors along the columns of the electrode (approx. 80% of total errors). Misinterpretation of the neighboring pads in the same row, across the midaxillary line, was substantially lower, with approx. 12% of total errors. Only approx. 8% of the total errors were between the non-neighboring pads along the electrode columns.

The confusion matrix for the movement pattern discrimination is shown in Figure 5b. The patterns from the zigzag and the side groups were relatively easy to recognize for the subjects, and the best result was achieved for the zigzag patterns. These patterns were well differentiated both within the group and between groups. However, the result was much lower for the letter group, where the subjects confused the two letters “u” and “n”, leading to 75% and 81% of correct recognitions of these two patterns, respectively.

### 3.2. Calibration

**Figure 6:**
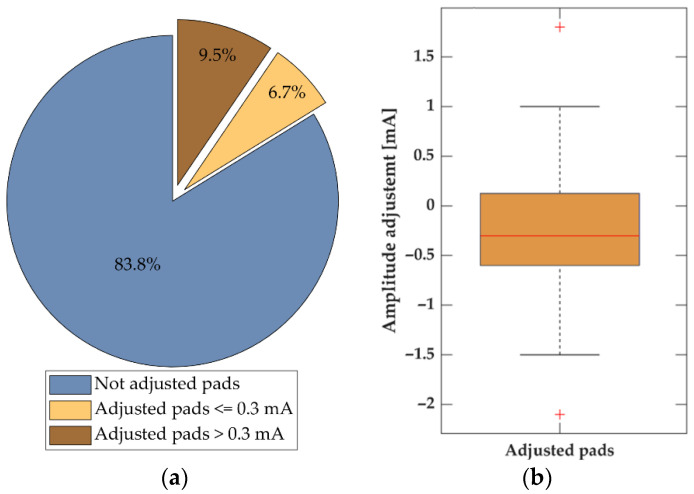
Manual adjustment of the automatically estimated localization amplitudes during the calibration process. (**a**) Pie chart indicating the percentage of pads not adjusted (blue), the percentage of pads with the amplitude adjusted, where the adjustment (absolute value) was less or equal to 0.3 mA (beige), and the percentage of pads with absolute adjustment greater than 0.3 mA (brown). (**b**) Boxplot of the difference between the automatically and manually adjusted pads amplitudes in cases when the amplitude was adjusted (16.2% of all pads).

The calibration procedure was of variable duration, lasting between 0.5 and 3 min, as the subjects could adjust the LTs manually in case the sensation was unpleasantly strong or not clear enough. In 9 out of 10 calibrations, the LA was not adjusted (83.8%) by the subjects (Figure 6a), or the amplitude was only changed by less than 0.3 mA (6.7%). Figure 6b shows boxplots of the magnitude of LA adjustments, i.e., the difference between automatically estimated LA and manually adjusted value. In most of the cases when the LA was changed, the LA value was decreased (74.3%). The median/IQRs were −0.3/0.7 mA. The previous results consider the adjustments at the level of individual pads. Considering calibration phases, in 16 out of 40 calibrations, the subjects used the option of additional amplitude adjustments and adjusted at least one pad. On average, 2.4 pads were adjusted in these cases.

## 4. Discussion

The present study showed that the subjects could achieve a high-accuracy recognition when discriminating between six electrotactile messages delivered through a matrix electrode placed on the torso using two encoding methods, namely, the static pads activation and the dynamic stimulation pattern approach (SR: 83.3% and 93.3%, Figure 4). The results suggest that the subjects were more successful when discriminating between six movement patterns compared to recognizing the six static pad activations, despite the identical stimulation time in both schemes (message duration). This is in line with earlier findings that reported better recognition rates when the dynamic approach to encode patterns was used, compared with a static approach, both in electrotactile [62] and vibrotactile [63] systems. Those findings indicate that dynamics patterns can be considered a better approach to achieve higher information bandwidth in tactile communication applications, even when conveying a relatively small set of messages (six in the present study) and systematically training the subjects in both encoding schemes. However, in both presented methods, four out of six messages achieved the SR > 85%, which is considered a high SR [7,64,65,66], indicating that the proposed system (electrode and encoding) is indeed a feasible communication channel.

During the spatial recognition test (Figure 5a), the pads facing the ventral side of the torso were on average better recognized compared to the pads facing the dorsal side (SR: 83.3% vs. 79.3%), which agrees with the findings in the literature that the ventral side of the torso is more sensitive and accurate [39,54]. The side of the body (i.e., front or back) was rarely misinterpreted, and most errors were made along the vertical axis of the electrode. We assume that the highest score for identification of the static stimuli on pad #6 was caused by the spatial acuity, which was assessed in the previous studies to be significantly lower and more sensitive at L4 than the T10 vertebral level on the back [67]. The spatial discrimination SR recorded in the current study is slightly higher than the SR reported in our former work, in which the SR of the six pads was 75% [55]. This increase in SR might be due to differences in the study design in which the static stimulation to the specific point was delivered twice and for a prolonged period (i.e., one time per 2 s [55] vs. two times per 3 s in the present study) to match the duration of the dynamic patterns.

In addition, the insights from the spatial discrimination tests seem to also be reflected in the recognition of the movement patterns. As explained in Section 2.3, the patterns were defined to incorporate two main cues that the subjects could exploit to identify the movement pattern. Namely, the side of the torso (ventral vs. dorsal pad activation) and the stimulation direction (upwards vs. downwards activation sequence). The high SR of the “side” group and the lower SR of the “letter” group indicate that the side of the torso was indeed more effective cue than the direction. “Side” patterns activated a single side, while the “letter” included both sides but different directions. However, the high SR of the “zigzag” group implies that comparable effectiveness can be ensured if both sides within the same pattern (as in letter) are activated alternately. The latter approach seems to be a particularly strong cue as this group resulted in the highest SR overall.

It should be noted that the used dynamic encoding scheme included activation of one electrode pad at a time, effectively “tracing” a shape onto the torso. The synchronous stimulation of more than one point was intentionally avoided as it can lead to more complex tactile sensations, depending on the stimulation parameters, especially intensity. For instance, two stimuli of low intensity may be felt as one positioned between the two active locations [68,69]. Alternatively, a stronger sensation on one site could mask the weaker one [70].

In the present study, the learning phase was short and lasted around five minutes. Nevertheless, a high SR was achieved with both encoding schemes, and this is encouraging result for the practical application of the system. Longer learning would likely lead to increased recognition rates, and thus higher bandwidth of tactile communication. The overall performance, however, would depend on the number of messages (six in the present study) but intuitive messages, in which the pattern corresponds to the message content (the meaning of the pattern), could facilitate the learning [2,71,72].

System usability is one of the key design objectives, and a fast system and calibration setup can be considered a requirement for resolving inherent drawbacks of the electrotactile stimulation approach. Due to the intrinsic human variability, the personalization of stimulation parameters is necessary to ensure a clear and comfortable perception of stimuli [73]. A critical factor for the applicability of an electrotactile interface is the speed of the calibration. The present study proposes a three-step approach: identifying the ST, estimating the LA (2 × ST) and potential manual readjustment of the estimated LA. The ST presents the minimum current amplitude eliciting sensation that can be perceived by the subject and therefore is the psychometric parameter that is the fastest to identify and it is unambiguous to both naïve and experienced subjects. The automatic LA estimation proposed in this study speeds up the calibration, however, only if the estimated value is not extensively readjusted by the subjects. For 9 out of 10 calibrations, the initially estimated LA was either not changed at all (83.8%) (Figure 6a) or required only a minor adjustment (considering 0.1 mA step, 0.3 mA is taken as a three click adjustment). Indeed, the experiments demonstrated that the proposed approach was successful, and hence a pad calibration was finished successfully for the majority of the trials after the second step. 

When the amplitude of the proposed LA was readjusted, most of the amplitudes were decreased (Figure 6b). The subjects mostly decreased the amplitude due to intensive sensation and muscle contraction. However, the median value of LA adjustments was rather small in most cases (median of 0.3 mA), and this indicates that the subjects adjusted the LA fast in only a few steps (~3 steps). The small size of the manual adjustment could indicate that the adjustment compensated for the error in ST identification, e.g., due to a slow reaction time of the subject. It should be noted that most subjects were naïve, without previous experience with electrotactile stimulation. The discomfort threshold is expected to increase with experience [74,75], which would in turn ensure less adjustments as the LA amplitude would be tolerated better in more cases. Although the LA values selected by the proposed procedure was clear and not uncomfortable, the perceived intensity is not guaranteed to be the same for every pad, which might be of importance in some use cases [4,27,76,77]. However, for the envisioned application, this observation is not a drawback. A relative change in the perceived stimulation intensity between pads would not confuse the subject but could rather be another factor the user may use in correctly identifying the message transmitted. Thus, we can conclude that the introduced calibration procedure and encoding methods are an important step towards fast and effective use of electrotactile communication by first responders. Although field trials will add another level of complexity and we can expect that feedback understanding during physical and cognitive load will decrease, the results of the tests in the static setup and controlled environment presented in this manuscript give us confidence that the presented system can, in the next step, be tested by the first responders in the relevant environment during activity.

## Data Availability

Not applicable.
